# Complete Genome Sequence of *Streptomyces* sp. TN58, a Producer of Acyl Alpha-l-Rhamnopyranosides

**DOI:** 10.1128/genomeA.00828-17

**Published:** 2017-08-24

**Authors:** Soumaya Najah, Teik Min Chong, Claude Gerbaud, Kok-Gan Chan, Lotfi Mellouli, Jean-Luc Pernodet

**Affiliations:** a Institute for Integrative Biology of the Cell (I2BC), CEA, CNRS, Université Paris-Sud, Université Paris-Saclay, Gif-sur-Yvette, France; b Laboratory of Microorganisms and Biomolecules of the Center of Biotechnology of Sfax (CBS), Sfax, Tunisia; c Division of Genetics and Molecular Biology, Institute of Biological Sciences, Faculty of Science, University of Malaya, Kuala Lumpur, Malaysia

## Abstract

*Streptomyces* sp. TN58, isolated from a Tunisian soil sample, produces several natural products, including acyl alpha-l-rhamnopyranosides. It possesses a 7.6-Mb linear chromosome. This is, to our knowledge, the first genome sequence of a microorganism known to produce acyl alpha-l-rhamnopyranosides, and it will be helpful to study the biosynthesis of these specialized metabolites.

## GENOME ANNOUNCEMENT

In the frame of a program aimed at finding new bioactive molecules for medicine and agriculture, *Streptomyces* sp. TN58 was isolated from a Tunisian soil sample (1). It was shown to produce five naturally bioactive compounds, including two acyl alpha-l-rhamnopyranosides (2) that possess biological activities of medical interest (3). Several acyl alpha-l-rhamnopyranosides produced by different *Streptomyces* species have been characterized (4, 5), but so far, their biosynthetic pathways have not been characterized. As *Streptomyces* sp. TN58 is amenable to genetic engineering (6), it constitutes a good model for studying acyl alpha-l-rhamnopyranoside biosynthesis.

A draft genome sequence of *Streptomyces* sp.TN58 was generated from paired-end libraries after sequencing using V2 Illumina sequencing chemistry (2- × 250-bp, 500-cycle kit) and a MiSeq instrument. The 26,879,362 Illumina reads were assembled with ABySS (7), yielding 79 contigs (coverage superior to 800-fold). The extremities of contigs often contained specialized metabolite biosynthetic genes, indicating that specialized metabolite biosynthetic gene clusters (BGCs) were probably split over several contigs. This hampered efficient mining of the genome. In order to obtain a complete genome, a run was performed with PacBio single-molecule real-time (SMRT) sequencing technology using two SMRT cells. It generated 154,892 reads with an average length of 9,420 nucleotides (nt). Assembly with the Hierarchical Genome Assembly Process 2 (8) yielded a single contiguous sequence of 7,399,903 bp (average coverage 137-fold). Comparison of the Illumina and PacBio assemblies readily identified small gaps, generally corresponding to 1 nucleotide missing in the PacBio sequence in a run of four or more Cs or Gs. We used the Illumina reads to correct the PacBio assembly. This was done with CLC Genomics Workbench 9 (Qiagen) and resulted in the insertion of 1,026 bases. The ends of the linear chromosomes of *Streptomyces* present terminal inverted repeats (TIRs) (9). Sequence comparisons and analysis performed as described previously (10) led us to consider that the first 193,941 bases of the sequence corresponded to one TIR, and a final chromosomal assembly of 7,585,034 bp was generated.

Gene prediction and annotation were performed using the NCBI Prokaryotic Genome Annotation Pipeline (https://www.ncbi.nlm.nih.gov/genome/annotation_prok/). This strain has a single chromosome with a G+C content of 72.3%. Seven rRNA operons, 69 tRNA genes, and 6,807 predicted coding sequences (CDSs) were found on the chromosome.

Pairwise average nucleotide identity (ANI) has been calculated for *Streptomyces* sp. TN58 and other *Streptomyces* type strains using the JSpecies Web server (11). ANI values above 99% have been obtained with the type strain *Streptomyces flavotricini* NRRL B-5419. These values, higher than the 95% to 96% threshold considered to be the bacterial species boundary (12), indicate that *Streptomyces* sp. TN58 belongs to the species *S. flavotricini*.

The potential of *Streptomyces* sp. TN58 to produce secondary metabolites was analyzed with antiSMASH4 (13). Twenty-seven specialized metabolite BGCs were detected. Concerning acyl alpha-l-rhamnopyranoside biosynthesis, no obvious candidate BGC was detected. However, the four genes involved in the biosynthesis of rhamnose, a precursor of acyl alpha-l-rhamnopyranoside, are present in *Streptomyces* sp. TN58. Genome sequence information will facilitate the study of the biosynthesis of acyl alpha-l-rhamnopyranosides.

### Accession number(s).

The chromosome sequence of *Streptomyces* sp. TN58 has been deposited in GenBank under the accession number CP018870. The version described in this paper is the first version, CP018870.1.
